# Broadly binding and functional antibodies and persisting memory B cells elicited by HIV vaccine PDPHV

**DOI:** 10.1038/s41541-022-00441-9

**Published:** 2022-02-09

**Authors:** Shixia Wang, Nicole L. Yates, Justin Pollara, Yegor Voronin, Sherry Stanfield-Oakley, Dong Han, Guangnan Hu, Wei Li, Guido Ferrari, Georgia D. Tomaras, Shan Lu

**Affiliations:** 1 University of Massachusetts Medical School, Worcester, MA USA; 2 Worcester HIV Vaccine Inc., Worcester, MA USA; 3 Department of Surgery, Duke University, Durham, NC USA

**Keywords:** Vaccines, Public health

## Abstract

Since publishing our original reports on the safety and immunogenicity of a polyvalent DNA prime-protein boost HIV vaccine (PDPHV) which elicited high titer antibody responses with broad specificity, neutralizing activities to multiple HIV-1 subtypes, as well as poly-functional T cell responses, accumulated findings from other HIV vaccine studies indicated the important roles of Ig isotype distribution, Fc medicated functions and the persistence of memory immune responses which were not studied in previous PDPHV related reports. The current report provides further detailed characterization of these parameters in human volunteers receiving the PDPHV regimen. Antibody responses were assessed using IgG isotype and gp70-V1V2-binding ELISAs, peptide arrays, and antibody-dependent cellular cytotoxicity (ADCC) assays. B cell ELISPOT was used to detect gp120-specific memory B cells. Our results showed that the gp120-specific antibodies were primarily of the IgG1 isotype. HIV-1 envelope protein variable regions V1 and V2 were actively targeted by the antibodies as determined by specific binding to both peptide and V1V2-carrying scaffolds. The antibodies showed potent and broad ADCC responses. Finally, the B cell ELISPOT analysis demonstrated persistence of gp120-specific memory B cells for at least 6 months after the last dose. These data indicate that broadly reactive binding Abs and ADCC responses as well as durable gp120-specific memory B cells were elicited by the polyvalent heterologous prime-boost vaccination regimens and showed great promise as a candidate HIV vaccine.

## Introduction

Development of a safe and effective vaccine is crucial for the control of the HIV pandemic. After the moderate success of the heterologous viral vector prime-protein boost approach in the RV144 trial in Thailand^1^, the HIV vaccine field continues to explore the various combinations of prime and boost modalities to improve the immunogenicity of preventive HIV vaccine candidates^2–5^.

Antibodies are known to be the key elements in vaccine-induced protection against a wide range of human infectious diseases, but the protective mechanisms are diverse. In recent years, it became clear that candidate HIV vaccines may elicit immune protection via Fc-mediated antibody functions^6–10^. In particular, detailed biomarker analysis of the RV144 trial showed that the gp70-V1V2-specific antibody responses and Fc-mediated antibody functions inversely correlated with the risk of infection while there were no broadly neutralizing antibodies (bNAbs) detected in protected volunteers^6,11^. This is an important finding because many previous HIV vaccine studies have only focused on bNAbs^12–14^.

The importance of Fc-mediated antibody functions was also reported from the study results in NHP models and HIV infected patients. ADCC responses were reported to inversely correlate with virus set point in acute SIV infection^15^ and in vaccinated animals following SHIV challenge^16,17^. In HIV-1 infection, a direct role for ADCC responses was shown in controlling virus replication by delaying overt disease^18–20^. HIV mother-to-child transmission (MTCT) studies also demonstrated that passively acquired Ab mediating ADCC responses could reduce mortality in HIV infected infants^21^, and higher pre-existing ADCC responses against exposure strains associated with less likelihood of HIV-1 MTCT and lower morbidity in infected infants^22^. Therefore, one of the major tasks in the HIV vaccine field is to further improve on the ADCC responses achieved by RV144^5^.

RV144 used the ALVAC prime-protein boost vaccine approach which belongs to the heterologous prime-boost strategy^23^. Another heterologous prime-boost approach is the DNA prime-protein boost which has been studied by our team in the last two decades^24–28^ including our first HIV vaccine clinical trial DP6-001^29^.

Using DNA-encoded gp120 immunogens to prime the host immune system with the matched gp120 proteins boost is a promising approach leading to high titers of functional antibodies and cell-mediated immune responses^29,30,31^. More importantly, the polyvalent antigen formulation has been shown effective in eliciting antibody responses across different clades of HIV-1 in the DP6-001 trial. The gp120-specific serum IgG responses were robust and broadly cross-reactive against gp120 antigens from a wide range of major HIV-1 clades and the neutralizing activities from volunteers’ immune sera were also cross-reactive against pseudotyped viruses expressing Env antigens from clades of A, B, C, and AE^29,31^. Furthermore, the mAbs isolated from DP6-001 volunteers showed broad binding to both autologous and heterologous Env antigens and mediated potent ADCC response^32^. Since the publication of initial reports on the overall immunogenicity of DP6-001 vaccine, much new information has been learned in the HIV-1 vaccine field such as the roles of IgG isotypes and the identification of V1V2 region as the possible target for ADCC responses^11,33^. In the current report, data from new studies are presented to provide more detailed assessment of the additional humoral responses including IgG isotypes, recognition profiles of HIV-1 Env V1V2 region, ADCC response and the gp120-specific peripheral memory B cell responses from these volunteers. By including the previously reported neutralizing antibody data^29,31^, the polyvalent DNA prime-protein boost regimen shows its potential to elicit poly-functional antibody responses against HIV-1.

## Results

### Sources of volunteer samples

The human samples used in the current studies were collected from the previously reported DP6-001 study which was a phase 1 clinical trial testing safety and immunogenicity of a polyvalent DNA-prime/protein-boost preventive HIV vaccine^29,30^. The DNA vaccine components included five plasmids encoding gp120 Env proteins from HIV-1 clades A, B, C, and AE (two variants used for clade B) and one plasmid encoding Gag protein from HIV-1 clade C. The protein vaccine includes five CHO-produced recombinant gp120 Env proteins that exactly match those expressed by the Env DNA vaccines, mixed with the QS-21 adjuvant at the time of injection. The volunteers received three doses of the DNA vaccine at weeks 0, 4, and 12 followed by protein boosts at weeks 20 and 28. Group A received the DNA vaccines intradermally and Group B intramuscularly (both in saline) without electroporation or other facilitated DNA delivery, while both groups received protein vaccines intramuscularly along with QS-21.

### IgG isotype analyses

We previously reported the dynamics of the overall gp120-binding IgG against the mixture of all five autologous gp120 proteins included in the vaccine formulation^29^. The individual total IgG titers at 2 weeks after the last immunization are shown in Supplementary Fig. 1. Both intradermal and intramuscular DNA priming immunizations resulted in median 2 × 10^5^ binding titers to the vaccine-matched gp120 proteins.

**Fig. 1:**
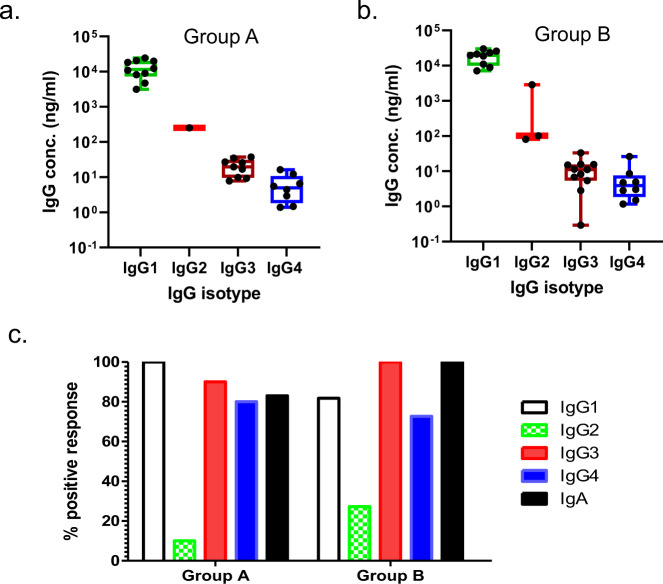
Env-specific IgG1, IgG2, IgG3 and IgG4 responses in DP6-001 volunteer sera. The magnitude of IgG isotype responses in volunteers receiving the DNA prime vaccine either intradermally (**a**) or intramuscularly (**b**). The response rates against IgG isotypes and IgA in volunteers from Groups A and B (**c**).

In the current study, we further explored the distribution of IgG isotypes elicited by the DP6-001 vaccine (Fig. 1a–c). Most volunteers were positive for IgG1, IgG3, and IgG4 antibodies against the consensus gp140-ConS antigen, while IgG2 antibodies were detected only in 10% (Group A) or 27% (Group B) of volunteers (Fig. 1c). When the magnitude of the response with each isotype was measured, the concentrations of IgG1 antibodies were found to be the highest among the four IgG isotypes and ranged between 10^3^ and 10^4 ^ng/mL (Fig. 1a, b). IgG3 and IgG4 concentrations were 100-1000 fold lower than IgG1 concentrations. Most of the vaccinees also developed gp120-specific IgA responses (Fig. 1c), but the magnitude of IgA responses was not determined.

### gp120 regions targeted by antibodies

**Fig. 2:**
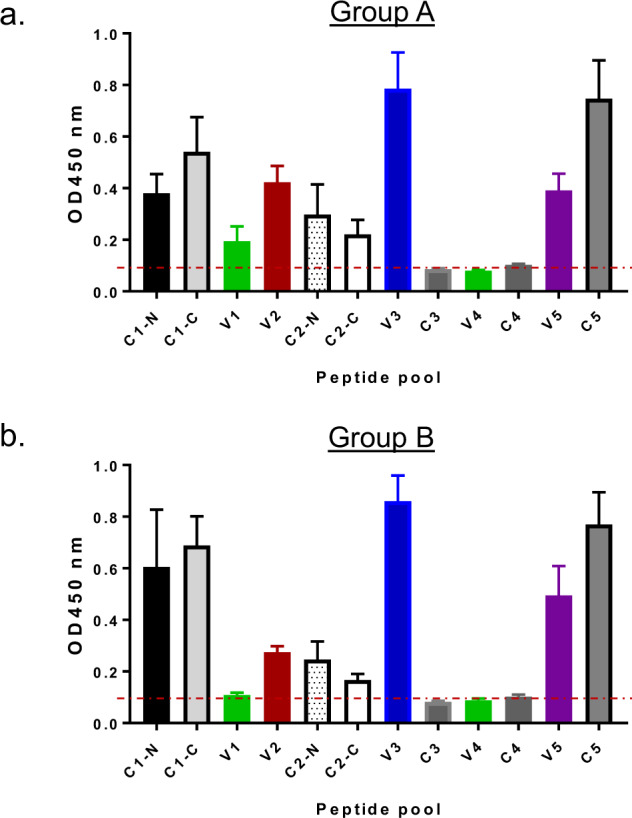
ELISA mapping of IgG responses in DP6-001 vaccinee sera (at 1:1000 dilution) against pooled overlapping peptides from different regions of Group B consensus gp120. Each bar represents the mean OD value of sera from either Group A (Panel **a**) or Group B (Panel **b**).

Next, we explored which regions of gp120 were primarily targeted by the serum antibodies elicited by vaccination. Binding to pools of linear peptides derived from each variable or conserved region of the consensus Group M gp120 protein was measured for each volunteer. Mean OD values at 1:1000 serum dilution for each group are shown in Fig. 2. Consistent with previous reports of gp120-elicited antibodies, variable region 3 (V3) was the dominant target for antibody responses, and constant regions 1 and 5 (C1 and C5) were also targeted. However, we also observed substantial binding to peptide pools derived from V1 and V2 regions, binding to which was previously identified as an inverse correlate of risk in the RV144 vaccine efficacy study^6,11,33^. There were no significant differences in antibody specificity between the two study groups (Fig. 2a and Fig. 2b).

**Fig. 3:**
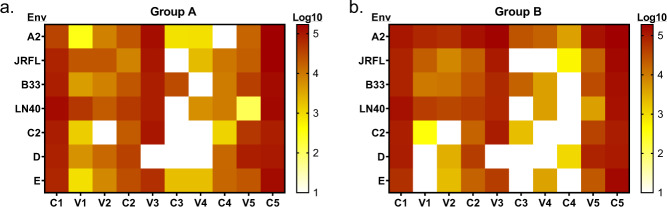
JPT microarray analysis of IgG specificity in DP6-001 vaccinee sera (Group A and Group B, at 1:1000 dilution) against different regions of individual gp120 proteins. The heatmap indicates mean binding intensity from Group A (Panel **a**) or Group B (Panel **b**) against each gp120 region. The side bar indicates the levels of responses.

To better dissect the specificity of antibody targeting and its relationship with a breadth of recognition of diverse HIV-1 strains, peptide arrays were created corresponding to seven strains covering the wide diversity of circulating HIV-1 (Fig. 3). Antibodies from sera of vaccinated volunteers bound to peptides from all seven strains in conserved regions C1, C2, and C5, while peptides from C3 and C4 were less effectively recognized, probably due to their internal position within the protein.

Among variable regions, peptides from V3 region were most broadly recognized, with clade D being the only non-recognized strain. Recognition of peptides from other variable regions was more variable and of lower magnitude than that of V3. Nevertheless, in V1 and V2 regions, sera from vaccinated volunteers bound to peptides from A2, JRFL, B33, and LN40 strains, as well as in some cases to peptides from C2, D, and E strains demonstrating the breadth of the response elicited by the polyvalent formulation of the vaccine. There is no significant difference between Group A and Group B (Fig. 3a and Fig. 3b).

**Fig. 4:**
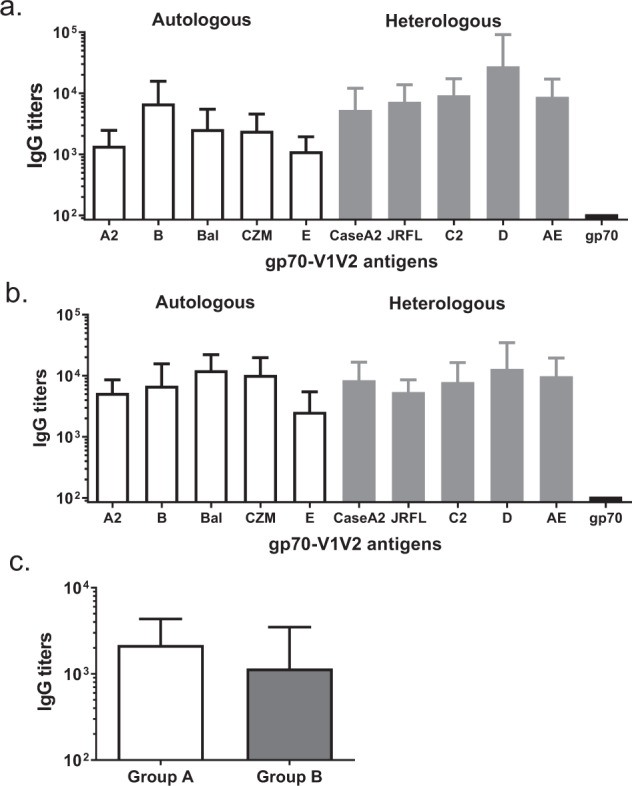
Detection of gp120 V1V2 specific antibody responses by ELISA. Antibody responses were measured against gp70 scaffold V1V2 antigens from either autologous gp120 or heterologous gp120 using sera from Group A (**a**) or Group B (**b**). Antibody responses against the cyclic V2 (A244) were also measured (**c**). Each bar represents the group mean titer with sera from each volunteer group.

The peptide-binding results indicated the breadth of V1V2 response in the vaccinees. In the inverse correlate of risk analysis of the RV144 study, the assay that identified V1V2 binding as a correlate relied on binding to the V1V2 region fused to a gp70 scaffold. Therefore, we explored the binding specificity of the sera against similar scaffolds carrying V1V2 regions from diverse strains of HIV-1 (Fig. 4a, b). When tested against scaffolds carrying V1V2 regions from the strains that were used in the vaccine (“autologous”), the binding was detected for all five V1V2-carrying scaffolds. The titers against reagents with V1V2 regions from strains not used in the vaccine (“heterologous”) were comparable to those against autologous V1V2 regions or in some cases were even higher. These results also demonstrated the high degree of breadth and magnitude of the responses elicited by polyvalent DP6-001 formulation.

Finally, we measured antibody binding to cyclic V1V2 region from Case-A2 strain, another reagent responses to which were identified as an inverse correlate of risk in the RV144 study, and found high binding titers (>10^3^) in both ID (Group A) and IM (Group B) DNA priming groups (Fig. 4c).

### Durability of gp120-specific memory B cell response

**Fig. 5:**
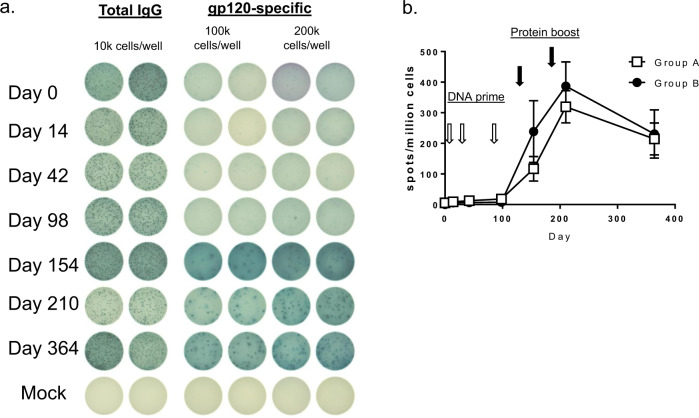
B cell ELISPOT was used to measure the gp120-specific memory B cell responses in DP6-001 volunteers. A mixture of five autologous gp120 proteins same as used in DP6-01 vaccine was coated on the plates. **a** ELISPOT readouts in duplicate from one volunteer sample collected at different time points of vaccination course are shown. **b** The dynamics of gp120-specific memory B cell responses is shown as the group average in spot/1 million cells with standard error.

Previous study demonstrated that the DP6-001 vaccine elicited durable antibody titers that only slightly declined over the 6-month period after the last vaccination^29^. To follow up on this finding we explored the development and dynamics of gp120-specific memory B cell response in the vaccinees. PBMC samples from pre-bleed, two weeks after each vaccination and at 6 months after the last dose were stimulated with interleukin-2 and a polyclonal activator, R848, to induce memory B cells to differentiate into antibody-secreting cells. Total antibody-secreting, as well as antigen-specific B cells, were then quantified by B cell ELISPOT (Fig. 5). DNA priming alone only minimally elicited gp120-specific memory B cells. However, after protein boost, gp120-specific memory B cells were greatly expanded with the peak of 300-400 cells per million PBMCs, which is consistent with the robust antibody response at those time points as reported^29^. After 6 months, the number of gp120-specific memory B cells decreased slightly but remained at ~200 cells per million PBMC, which indicated a long-term B cell memory after the initial response to vaccination.

### ADCC responses

**Fig. 6:**
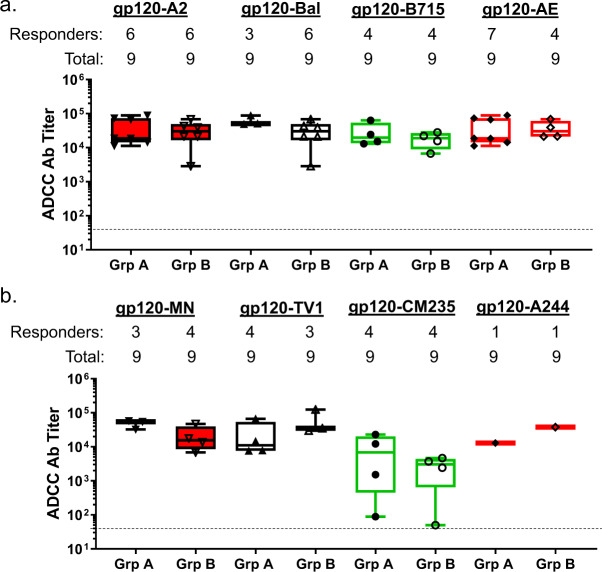
Rate and magnitude of ADCC response against gp120-coated target cells in DP6-001 volunteer sera. ADCC-GTL assay was to detect ADCC against target cells coated either with the autologous gp120 proteins (**a**) or with the heterologous gp120 proteins (**b**). The numbers of positive responders and total volunteers are shown above the plots. The cut-off line is shown and the negative responders below the cut-off level were not included in the magnitude analysis.

We first assessed ADCC response against target cells coated with various HIV-1 gp120 proteins (Fig. 6 and Supplementary Table 1). When tested against the gp120 proteins autologous to the gp120 antigens used as part of the DNA/protein vaccine formulation, we observed the highest response rate against cells coated with clade AE gp120 (93TH976) (7 out of 9 tested plasma samples from Group B and 8 out of 9 from Group A) followed by the response rate against the clade A gp120 (92UG037) (6 out of 9 tested serum samples for both Groups A and B). The response rate against the other two autologous clade B gp120 proteins (B715 and Bal) was lower, with three and five volunteers having a response, respectively.

When tested against the gp120 proteins heterologous to the gp120 antigens used in the vaccine formulation, the ADCC response rate was overall lower with 3 or 4 volunteers out of 9 showing a positive response to gp120 antigens from clades B (MN), C (TV1), and AE (CM235). Lastly, only one Group A and one Group B vaccine recipient had detectable ADCC response heterologous clade AE gp120 antigen (A244) (Supplementary Table 1).

When positive samples were considered, the magnitude of ADCC responses was high against A2, B715, and Bal (autologous, Fig. 6a) and MN and TV1 (heterologous, Fig. 6b) with mean titers over 1:10,000. ADCC Ab titers below 1:10,000 were detectable against AE (autologous, Fig. 6a) and CM235 (heterologous, Fig. 6b). In general, there is no significant magnitude difference between Group A and Group B, except for ADCC activities against CM235 which showed a high degree of variation among serum samples from volunteers in Group A but minimal variation in volunteers from Group B (Fig. 6b).

**Fig. 7:**
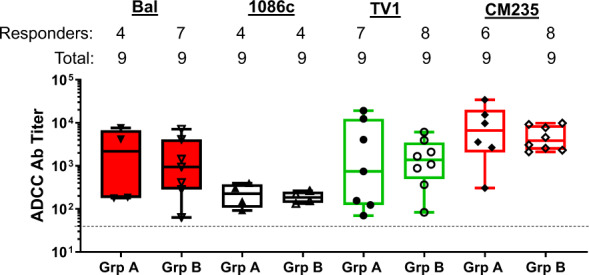
Rate and magnitude of ADCC response against infectious molecular clone (IMC) infected target cells in DP6-001 volunteer sera. ADCC-Luc assay was used to detect ADCC against target cells infected with one of the four IMCs from HIV-1 clade B (Bal), C (1086c and TV1), and AE (CM235). The cut-off line is shown and the negative responders below the cut-off level were not included in the magnitude analysis.

Similar studies were done to measure ADCC activities against target cells infected by infectious molecular clones (IMC) from clades B (Bal-IMC), C (TV1-IMC, 1086c-IMC), and AE (CM235-IMC), all of them different from the viral variants included in the vaccine. In this study, IL-15-treated PBMCs were used as effector cells (33). The frequency of positive ADCC responses against all four IMCs were shown in Supplementary Table 2. The ADCC response rate was >44% (4–8 serum samples out of 9 serum samples tested (Fig. 7)). The ADCC Ab titers against IMC infected cells ranged from 1:100 to close to 1:10,000. There was no difference in positive response rates or magnitude of responses between Group A and Group B.

For both ADCC against gp120-coated target cells and ADCC against IMC infected target cells, negative responders with ADCC activities below cut-off were not included for magnitude analysis.

## Discussion

The current report expands the previous findings of high antibody titers elicited in phase 1 clinical trial DP6-001 of the polyvalent DNA prime-protein boost vaccine by demonstrating the breadth of these responses and their functionality.

We show that elicited antibodies have binding characteristics that have previously been identified as protective in an efficacy study RV144^1,6,11,33^. Specifically, V1V2 peptides and V1V2-based reagents from a wide diversity of HIV-1 clades were recognized by vaccinee sera. Our previously published data showed that the same vaccinee sera have neutralization activity against a wide range of HIV-1 variants from diverse clades^29^. These results indicate that antibodies elicited by DP6-001 formulation are able to recognize broad HIV-1 envelopes.

The new IgG isotype analysis included in the current report demonstrated that the majority of antibody responses are of IgG1 isotype, the predominant isotype observed in response to viral infections and vaccines. Our study also showed a high rate of IgG3 responses similar to the rate of IgG1 responses, however, the titers of IgG3 were lower than those for IgG1. Both IgG1 and IgG3 are known to have a high affinity for FcγRs and are potent activators of Fc-mediated functional activity. Literature showed that antibodies directed against the V1V2 region of gp120, in particular the IgG1 and IgG3 subclass mediating ADCC response, seem to play a predominant role in protection against HIV-1 acquisition^34^. On the other hand, the response rate of IgG2 and IgG4 were low in our study, similar to what were observed in other studies.

In the current report, we measured total IgG responses against V1V2, but not for different subtypes of Ig. The measurement of IgG3 against V1V2 could be performed in future studies. The rate of IgA responses among both study groups were also measured but not for their magnitude. We didn’t analyze the ADCC with IgA levels in the current study. The competition of IgA with ADCC responses in RV144 was demonstrated using monoclonal Ab. It is difficult to adjust the concentration of polyclonal IgA to compete IgG functions without assessing the specificity of the responses.

In recent years, based on results from both human and animal studies it became clear that candidate HIV-1 vaccines may elicit immune protection via Fc-mediated antibody functions^6–10^. In particular, detailed biomarker analysis of RV144 trial and HVTN 505 showed that Fc-mediated antibody functions inversely correlated with the risk of infection in this trial^6,11,35^ and with viral load set point^35^. In the current study, potent and broad ADCC activity was shown in DP6-001 immune sera, a response that was shown inversely correlated with the risk of infection in the RV144 study.

Our study demonstrated that DP6-001 volunteer sera had ADCC responses against IMC infected targeted cells while most of the previous HIV-1 vaccine studies were mainly directed ADCC against gp120-coated target cells^36^. Presumably, ADCC against IMC infected cells would target more on conformational epitopes while ADCC against gp120-coated targes may aim at CD4-induced epitopes^7,37,38^.

The broad reactivity observed in neutralizing antibodies^29^, gp120-binding, V1V2-binding, and ADCC assays supports the concept of polyvalency for HIV-1 vaccine, an approach that has not been sufficiently explored in the HIV vaccine field. The polyvalent formulation studied here resulted in responses that go beyond the vaccine-autologous strains and extend to diverse variants from different HIV-1 clades.

Finally, we demonstrate that gp120-specific memory B cells were generated in the course of vaccination and these cells persisted in vaccinated volunteers for at least 6 months after the last vaccination. These long-lasting, high-level memory responses elicited by the PDPHV regimen may not need a late boost as had been conducted in the RV305 study. This finding is consistent with the previously described sustained antibody titers^29^. High level and persisting antigen-specific memory B cell responses should be the results of effective germinal center B cell activation stimulated by DNA immunization^39^. In line with this finding, we previously reported that DNA immunization can improve the production of high-affinity antibodies^40^. While the HVTN lab published the ELISPOT method to monitor B cells in HIV-1 infected patients^41^, the current report would be the first to show the persistent Env-specific memory B cell responses induced by an HIV-1 vaccine.

The DP6-001 is the first-in-human study of the polyvalent DNA prime protein-boost vaccine^29^. The results indicate that this approach is able to induce humoral responses with high response rates, high titers and breadth, functional significance, and long durability. Manufacturing polyvalent DNA and protein vaccines may generate certain programmatic challenges, but the vaccine industry is very experienced in producing polyvalent vaccines. Several new batches of our PDPHV were manufactured in the last few years. The second generation of this vaccine has been recently tested in the HVTN 124 trial by the HIV Vaccine Trials Network which will provide further information about this approach.

## Methods

### Serum and PBMC samples from DP6-001 vaccinees

The serum and PBMC samples were from a closed phase I trial DP6-001 (NCT00061243)^29^. All participants gave written informed consent. The serum and PBMC samples were collected according to protocols approved by the institutional review board (IRB) at the University of Massachusetts Medical School (UMMS), USA^29^

Groups A or B of DP6-001 received three DNA immunizations at Days 0, 28, and 84, by either ID or IM needle injections, respectively, with a dose of 1.2 mg DNA containing five gp120 and one Gag plasmids formulated in 0.2 ml saline as previously reported^29^. Protein boosts contained a fixed dose of five recombinant gp120 proteins (0.375 mg) with adjuvant QS-21 administered twice at Days 140 and 196. The 5-valent gp120 recombinant proteins matched the polyvalent gp120 DNA vaccines. The serum samples used in the current report were collected on Day 0 and Day 210. The PBMC samples used in this study were collected on Day 0 and 14 days after each immunization, and at the study closeout.

### Proteins and peptides

Recombinant gp120, and gp70-V1V2 proteins and peptides were used to evaluate the Env-specific antibody and B cell responses, including the autologous gp120 proteins from clades A (92UG037, A2), B (92US715, B715, and Bal), C (96ZM651, CZM), and AE (93TH976), and the heterologous gp120 proteins from clades B (MN), C (TV1) and AE (CM235 and A244); and gp140-ConS. Ten gp70-V1V2 proteins covered HIV-1 clades A (92UG037), B (92US715, Bal, JRFL, and Case-A2), C (96ZM965 and 93MW965.26), D (92UG021.16), and AE (93TH976 and consensus), and gp70 backbone protein was included as a negative control.

The Group M consensus gp120 peptides (15-mer with 11-overlap) were from NIH AIDS Reagent Program. Twelve overlap peptide pools were prepared to cover each gp120 region for ELISA assays: C1-N (14 peptides), C1-C (14 peptides), V1 (7 peptides), V2 (11 peptides), C2-N (11 peptides), C2-C (11 peptides), V3 (10 peptides), C3 (10 peptides), V4 (7 peptides), C4 (10 peptides), V5 (7 peptides) and C5 (6 peptides). The gp120 peptides printed on microarray slides were 15-mer with 11-overlap generated by JPT Peptide Technologies (Berlin, Germany) covering HIV-1 clade A (92UG037), B (JRFL, B33, LN40^42^), C (93MW965), D (92UG021), and AE (consensus). The cyclic V2 peptide (CSFNMTTELRDKKQKVHALFYKLDIVPIEDNTSSSEYRLINC) for clade AE (A244)^33^ was synthesized by EZBiolab (Carmel, IN).

### Enzyme-linked immunosorbent assay (ELISA)

ELISAs were performed in 96-well microtiter plates. For gp120-specific antibody detection, the plates were coated with the mixture of five autologous gp120 antigens (92UG037, 92US715, Bal, 96ZM651, and 93TH976) at 1μg/ml in PBS (100 μL) at room temperature (RT) for 1 h. For gp70-V1V2-specific antibody detection, the plates were coated with individual gp70-V1V2 protein at 1 μg/ml in PBS (100 μL) at RT for 1 h. For detection of peptide-specific antibody responses, the plates were coated with peptides at 5 μg/mL at 4 °C overnight. After the relevant antigen coating, the ELISA was performed following previously established protocols^29,31,43^.

### Linear epitope mapping by peptide microarray

The sera collected at 2 weeks after the second protein boost and pre-bleed as control were analyzed against a library of HIV-1 Env linear peptides using JPT microarray^43^. Briefly, the peptide microarray was performed using a Tecan HS4000 Hybridization WorkStation. Arrays were scanned using an Axon GenePix 4300 Scanner (Molecular Devices, Sunnyvale, CA). Images were analyzed using GenePix Pro 7 software (Molecular Devices).

### Isotype binding antibodies

The Ig isotyping of Env-specific antibody responses in DP6-001 vaccinee sera was performed similarly to previously described^11,44,45^. HIV Env ConS gp140 antigen (Drs. Liao and Haynes, Duke University), purified IgG, IgG1, IgG2, IgG3, IgG4, and IgA proteins (Sigma-Aldrich, St. Louis, MO, USA, used as controls) were covalently coupled to carboxylated xMAP microspheres. HIV-specific antibody subclasses in serum samples were detected with biotin-conjugated mouse anti-human isotype antibodies. Detailed information can be found in the above references. All assays were run under Good Clinical Laboratory Practice compliant conditions.

### Memory B cell ELISPOT

PBMCs from DP6-001 vaccinee was first treated to induce memory B cells to differentiate into antibody-secreting cells by a 5-day stimulation, similar to previous report^41^. Briefly, the PBMCs were cultured at 1 × 10^6^ cells/ml in R-10 supplemented with 1 μg/ml R848 (Mabtech, Cincinnati, OH) and 10 ng/ml human IL-2 (Mabtech). The 96-well ELISPOT filter plates (Millipore) were coated with the mixture of five autologous gp120 antigens (A2, B715, Bal, Czm, and AE) at 10 μg/ml in PBS (100 μL) or goat anti-human IgG (Mabtech) at 4 °C overnight. After blocking, the stimulated PBMCs were added to the plates at 0.1 and 0.2 million cells/well, and incubated for 16 h. Plates were subsequentially incubated with biotinylated anti-human-IgG (Mabtech) for 2 h at RT, Streptavidin-ALP conjugate (Mabtech) for 1 h, and then developed using BCIP/NBT-plus substrate. The plates were washed with PBS between steps. The number of spots was scanned and analyzed using an automated ELISPOT counter.

### ADCC assays

The HIV-specific antibody-dependent cell-mediated cytotoxicity (ADCC) activities in DP6-001 sera were evaluated using two types of assays that have been qualified for testing samples from clinical trials and detect responses related to two immunological spaces:^36^ (1) GranToxiLux (GTL)-ADCC assay against gp120 protein-coated target cells^46,47^, and (2) Luciferase-based ADCC (ADCC-Luc) assay against HIV-1 infectious molecular clone (IMC) infected target cells^48^. FACS gating strategy is provided in Supplementary Fig. 2.

The ADCC‐GTL assay was performed based on well-established protocols^46,47^. The gp120-coated CEM.NKR_CCR5_ CD4^+^ T cell line was used as the target cells. Briefly, individual gp120 protein-coated target cells were labeled with TFL4 and NFL1 (both from OncoImmunin, Gaithersburg, MD). Then, 10^4^ target cells per well were added to 96‐well V‐bottom plates and incubated with the Granzyme B (GzB) substrate (OncoImmunin) and effector cells for 5 min at RT. Effector cells were PBMC obtained from an HIV-seronegative donor heterozygous for FcγR3A at position 158 (158 F/V) and for FcγR2A at position 131 (131H/R)^49–51^. PBMC was obtained by leukapheresis to collect enough cells for completion of the study with a single donation^52^, minimizing any potential for variability in the effector cell populations to influence the study outcome. PBMC were used at an effector cell to target cell ratio of 30:1. The antibodies were then added and tested after four-fold serial dilutions starting at 1:100; the plates were incubated an additional 15 min at RT, then for 1 h at 37 °C 5% CO_2_ following centrifugation for 1 min at 300 g. The plates were then incubated for 30 min at 4 °C and subsequently washed three times with 1% FBS PBS wash buffer. Well contents were then re‐suspended in 150 µl wash buffer and acquired directly with BD Fortessa flow cytometer (BD Biosciences, San Jose, CA) within 4 h using the High Throughput Sampler (HTS, BD Biosciences). Flow cytometry data analysis was performed using FlowJo 9.9.4 software (FlowJo, LLC., Ashland OR). Data are reported as the maximum proportion of cells positive for proteolytically active granzyme B (GzB) out of the total viable target cell population (maximum %GzB activity) after subtracting the background activity observed in wells containing effector and target cells in the absence of plasma. ADCC endpoint titers were determined by interpolating the last sera dilution above the previously established positive cutoff for this assay (8% GzB activity) using GraphPad Prism, version 7.0b software (GraphPad Software, Inc., La Jolla, CA) and were reported as reciprocal dilution.

ADCC activity was also determined by a luciferase (Luc)‐based assay based on well-established protocols^47,48^. CEM.NKRCCR5 target cells infected with HIV‐1 IMC encoding Renilla luciferase were incubated with PBMC obtained by leukapheresis to collect enough cells for completion of the study with a single donation as described for the GTL assay. PBMC effector cells treated or untreated with IL-15 and antibodies in ½ area opaque flat-bottom plates for 30 min at RT in duplicate wells. The plates were then centrifuged for 1 min at 300 g and subsequently incubated for an additional 5.5 h at 37 °C with 5% CO_2_. ADCC activity, reported as percent specific killing, was calculated from the change in Relative Light Units (RLU; ViviRen luciferase assay; Promega, Madison, WI) resulting from the loss of intact target cells in wells containing effector cells, target cells, and antibody samples compared to amounts in control wells containing target cells and effector cells alone according to the following formula: percent specific killing = [(number of RLU of target and effector well—number of RLU of the test well)/number of RLU of target and effector well] × 100. ADCC endpoint titers were determined by interpolating the last sera dilution above the positive cut-off for this assay (10% specific killing) using GraphPad Prism, version 7.0b software after subtracting the background activity observed for matched pre-vaccination samples, and were reported as reciprocal dilution.

### Statistical analyses

One-way ANOVA was used to compare the average natural log-transformed Env-specific antibody magnitudes, IgG isotype concentrations, ADCC titers, and the number of B cells between groups A and B. The *p-*value < 0.05 (*p* < *0.05*) was considered as a significant difference.

### Reporting summary

Further information on research design is available in the Nature Research Reporting Summary linked to this article.

## Supplementary information

Supplementary information

REPORTING SUMMARY

## Data Availability

The data that support the findings from this study are available from the corresponding author on reasonable request.
