# Supplementary material for: Broadly binding and functional antibodies and persisting memory B cells elicited by HIV vaccine PDPHV
Source: NPJ Vaccines. 2022 Feb 9;7:18. doi: 10.1038/s41541-022-00441-9 (PMC8828892; doi:10.1038/s41541-022-00441-9)
Supplement: Supplementary file 1 — Supplementary information [file 41541_2022_441_MOESM1_ESM.pdf]

Supplementary Table 1

ADCC antibody titers against gp120- coated target cells in individual subjects

Target	gp120 coated target cells							
Clade	A	B			C	AE		
Isolate	92UG037 (A2)	92US715 (B)	Bal	MN	TV1	93TH976 (AE)	CM235	A244
Group A								
ABL002	9857.1	-	-	32514.6	-	11276.4	1523.8	-
ABL005	-	-	-	-	-	14323.3	-	-
ABL007	13910.9	24195.3	-	-	14424.7	18664.8	-	-
ABL009	*ND*	*ND*	>10,000	*ND*	*ND*	>10,000	*ND*	*ND*
ABL013	26604.4	12979.8	49140.0	54179.6	7752.9	73526.7	-	-
ABL017	-	-	-	-	-	-	-	-
ABL021	6647.1	15110.3	87702.7	-	66678.1	66345.7	12157.0	-
ABL022	92985.6	63666.3	50776.8	57737.9	7588.2	88752.8	22864.8	12893.5
ABL030	31710.6	-	-	-	-	18496.2	89.0	-
ABL034	ND	*ND*	>10,000	*ND*	*ND*	>10,000	*ND*	*ND*
ABL035	-	-	-	-	-	-	-	-
Group B								
ABL001	*ND*	*ND*	>10,000	*ND*	*ND*	>10,000	*ND*	*ND*
ABL003	*ND*	*ND*	>10,000	*ND*	*ND*	>10,000	*ND*	*ND*
ABL010	14371.4	28117.7	28164.9	47150.7	35127.5	39087.4	3695.1	-
ABL015	-	-	-	-	-	-	-	-
ABL016	34666.6	22149.8	64703.0	17622.8	29845.5	68204.9	2432.7	37907.6
ABL020	6301.2	-	23606.4	-	124249.0	21509.0	50.0	-
ABL023	23759.4	6705.5	18814.7	13605.5	-	21206.1	4653.0	-
ABL024	43148.5	15598.5	12104.8	6834.6	-	-	-	-
ABL029	-	-	-	-	-	-	-	-
ABL031	2868.3	-	-	-	-	-	-	-
ABL033	-	-	-	-	-	-	-	-
Negative control								
ABL012	-	-	-	-	-	-	-	-

Supplementary Table 2

ADCC antibody titers in against IMC infected cells in individual subjects

Target	HIV-1 infectious clone infected target cells			
Clade	B	C		AE
Isolate	Bal-IMC	TV1-IMC	1086c-IMC	CM235-IMC
Group A				
ABL002	186.0	742.6	149.4	2,657.2
ABL005	-	69.9	-	304.7
ABL007	7,626.2	12,335.0	395.7	15,267.8
ABL009	*ND*	*ND*	*ND*	*ND*
ABL013	-	156.0	-	3,551.0
ABL017	-	123.8	92.4	-
ABL021	4,158.0	4,070.1	-	9,734.1
ABL022	177.9	19,162.8	303.9	34,161.9
ABL030	-	-	-	-
ABL034	*ND*	*ND*	*ND*	*ND*
ABL035	-	-	-	-
Group B				
ABL001	*ND*	*ND*	*ND*	*ND*
ABL003	*ND*	*ND*	*ND*	*ND*
ABL010	4,111.0	6,080.6	217.6	9,175.9
ABL015	-	-	-	-
ABL016	7,118.6	3,940.5	-	7,710.2
ABL020	945.2	883.0	-	2,317.6
ABL023	-	83.2	-	4,585.3
ABL024	412.7	1,669.6	133.1	2,564.7
ABL029	281.9	364.6	154.3	2,119.1
ABL031	63.3	1,084.6	-	3,082.2
ABL033	1,438.0	2,100.5	264.3	9,817.9
Placebo control				
ABL012	-	-	-	-

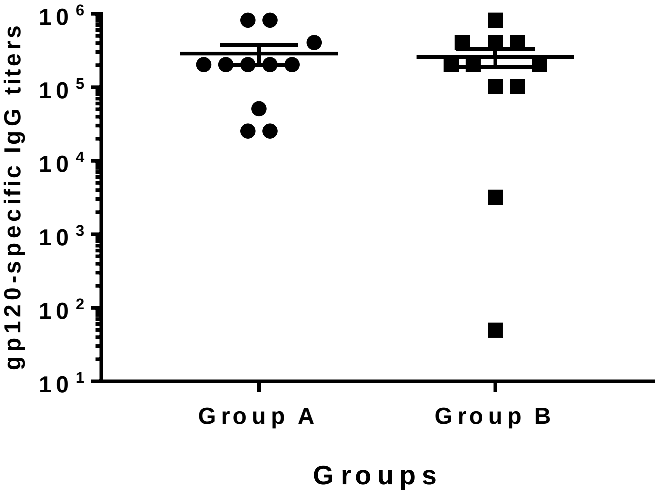

Supplementary Figure 1

The gp120 specific antibody titers in DP6-001 volunteers at two weeks after the 2nd protein boost. The autologous 5-gp120 protein mixture was used to coat the ELISA plates. Each dot indicates one individual subject in Groups A or B.

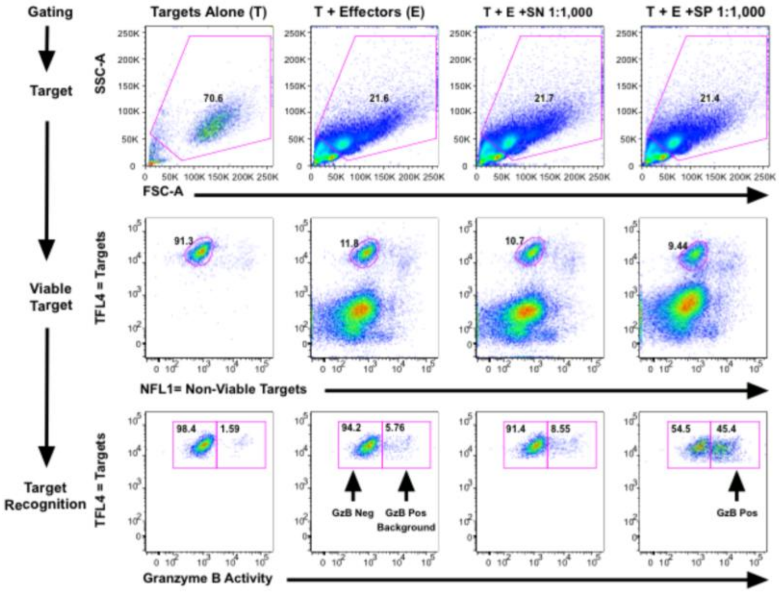

Supplementary Figure 2

Gating strategy used to measure rate and magnitude of ADCC response in Fig 6 and Fig 7 is described here. Using target cells alone, the first gate was set to include total target cells (top row of panel). The identification of the viable target cells is performed by combining the analysis of the signal emitted by the TFL4 (separation of target from effector cells) and NFL1 (separation of viable NFL1 negative from dead NFL1 positive cells) as reported in the dot plots in the middle row. Lastly, the GzB positive cells are identified as those that are outside the GzB negative gate set using the gate from the target in absence of the effectors (first left dot plot, bottom row). The frequency of the GzB positive population in the condition where target and effector cells were incubated without serum/plasma was considered as our background activity. More details of ADCC assay and FACS gating are available in reference #46.
