# Supplementary material for: Broadly binding and functional antibodies and persisting memory B cells elicited by HIV vaccine PDPHV
Source: NPJ Vaccines. 2022 Feb 9;7:18. doi: 10.1038/s41541-022-00441-9 (PMC8828892; doi:10.1038/s41541-022-00441-9)
Supplement: Supplementary file 2 — REPORTING SUMMARY [file 41541_2022_441_MOESM2_ESM.pdf]

nature portfolio

Corresponding author(s): Shan Lu

Last updated by author(s): Nov 30, 2021

# Reporting Summary

Nature Portfolio wishes to improve the reproducibility of the work that we publish. This form provides structure for consistency and transparency in reporting. For further information on Nature Portfolio policies, see our Editorial Policies and the Editorial Policy Checklist.

## Statistics

For all statistical analyses, confirm that the following items are present in the figure legend, table legend, main text, or Methods section.

| n/a | Confirmed | |
|---|---|---|
| ☐ | ☒ | The exact sample size ($n$) for each experimental group/condition, given as a discrete number and unit of measurement |
| ☒ | ☐ | A statement on whether measurements were taken from distinct samples or whether the same sample was measured repeatedly |
| ☒ | ☐ | The statistical test(s) used AND whether they are one- or two-sided<br>*Only common tests should be described solely by name; describe more complex techniques in the Methods section.* |
| ☒ | ☐ | A description of all covariates tested |
| ☒ | ☐ | A description of any assumptions or corrections, such as tests of normality and adjustment for multiple comparisons |
| ☒ | ☐ | A full description of the statistical parameters including central tendency (e.g. means) or other basic estimates (e.g. regression coefficient) AND variation (e.g. standard deviation) or associated estimates of uncertainty (e.g. confidence intervals) |
| ☒ | ☐ | For null hypothesis testing, the test statistic (e.g. $F$, $t$, $r$) with confidence intervals, effect sizes, degrees of freedom and $P$ value noted<br>*Give P values as exact values whenever suitable.* |
| ☒ | ☐ | For Bayesian analysis, information on the choice of priors and Markov chain Monte Carlo settings |
| ☒ | ☐ | For hierarchical and complex designs, identification of the appropriate level for tests and full reporting of outcomes |
| ☒ | ☐ | Estimates of effect sizes (e.g. Cohen's $d$, Pearson's $r$), indicating how they were calculated |

*Our web collection on statistics for biologists contains articles on many of the points above.*

## Software and code

Policy information about availability of computer code

| | |
|---|---|
| Data collection | N/A |
| Data analysis | N/A |

For manuscripts utilizing custom algorithms or software that are central to the research but not yet described in published literature, software must be made available to editors and reviewers. We strongly encourage code deposition in a community repository (e.g. GitHub). See the Nature Portfolio guidelines for submitting code & software for further information.

## Data

Policy information about availability of data

All manuscripts must include a data availability statement. This statement should provide the following information, where applicable:

- Accession codes, unique identifiers, or web links for publicly available datasets
- A description of any restrictions on data availability
- For clinical datasets or third party data, please ensure that the statement adheres to our policy

*Provide your data availability statement here.*

# Field-specific reporting

Please select the one below that is the best fit for your research. If you are not sure, read the appropriate sections before making your selection.

☒ Life sciences ☐ Behavioural & social sciences ☐ Ecological, evolutionary & environmental sciences

For a reference copy of the document with all sections, see nature.com/documents/nr-reporting-summary-flat.pdf

# Life sciences study design

All studies must disclose on these points even when the disclosure is negative.

| | |
|---|---|
| Sample size | Based on available number of samples |
| Data exclusions | No data exclusion |
| Replication | Key studies were repeated to confirm the reproducibility |
| Randomization | N/A |
| Blinding | N/A |

# Reporting for specific materials, systems and methods

We require information from authors about some types of materials, experimental systems and methods used in many studies. Here, indicate whether each material, system or method listed is relevant to your study. If you are not sure if a list item applies to your research, read the appropriate section before selecting a response.

## Materials & experimental systems

| n/a | Involved in the study | |
|---|---|---|
| ☐ | ☒ | Antibodies |
| ☒ | ☐ | Eukaryotic cell lines |
| ☒ | ☐ | Palaeontology and archaeology |
| ☒ | ☐ | Animals and other organisms |
| ☒ | ☐ | Human research participants |
| ☒ | ☐ | Clinical data |
| ☒ | ☐ | Dual use research of concern |

## Methods

| n/a | Involved in the study | |
|---|---|---|
| ☒ | ☐ | ChIP-seq |
| ☐ | ☒ | Flow cytometry |
| ☒ | ☐ | MRI-based neuroimaging |

## Antibodies

| | |
|---|---|
| Antibodies used | 1. Goat Anti-Human IgG Antibody (H+L), Biotinylated, Vector Lab (Cat# BA-3000-1.5)<br>2. Goat anti-human IgG, biotinylated, MabTech (Cat# 3820-4-250) |
| Validation | 1. Anti Human IgG Antibody (H+L), made in goat, Biotinylated x human - https://vectorlabs.com/biotinylated-goat-anti-human-igg-antibody.html<br>2. Goat anti-human IgG, biotinylated (3820-4-250) - https://www.mabtech.com/products/goat-anti-human-igg-biotinylated-3820-4 |

## Flow Cytometry

### Plots

Confirm that:

☐ The axis labels state the marker and fluorochrome used (e.g. CD4-FITC).

☐ The axis scales are clearly visible. Include numbers along axes only for bottom left plot of group (a 'group' is an analysis of identical markers).

☐ All plots are contour plots with outliers or pseudocolor plots.

☒ A numerical value for number of cells or percentage (with statistics) is provided.

### Methodology

| | |
|---|---|
| Sample preparation | The gp120 coated CEM.NKRCCR5 CD4+ T cell line was used as the target cells. 104 target cells per well were added to 96- |

| | |
|---|---|
| Sample preparation | well V-bottom plates and incubated with the Granzyme B (GzB) substrate (OncoImmunin) and effector cells for 5 min at RT. Effector cells were PBMC obtained from a HIV-seronegative donor heterozygous for Fc?R3A at position 158 (158F/V) and for Fc?R2A at position 131 (131H/R). PBMC were used at an effector cell to target cell ratio of 30:1. |
| Instrument | BD Fortessa flow cytometer (BD Biosciences, San Jose, CA) |
| Software | FlowJo 9.9.4 software (FlowJo, LLC., Ashland OR) |
| Cell population abundance | The maximum proportion of cells positive for proteolytically active granzyme B (GzB) out of the total viable target cell population (maximum %GzB activity) after subtracting the background activity observed in wells containing effector and target cells in the absence of plasma. |
| Gating strategy | FSC/SSC gates of the starting cell population, to gate viable cells; then, gate TFL4 positive target cells; finally gate GzB positive and negative cells, to calculate % GzB positive cells. |

☐ Tick this box to confirm that a figure exemplifying the gating strategy is provided in the Supplementary Information.
